# A Miniaturized 3D Heat Flux Sensor to Characterize Heat Transfer in Regolith of Planets and Small Bodies

**DOI:** 10.3390/s20154135

**Published:** 2020-07-25

**Authors:** Manuel Domínguez-Pumar, Jose-Antonio Rodríguez-Manfredi, Vicente Jiménez, Sandra Bermejo, Joan Pons-Nin

**Affiliations:** 1 Micro and Nano Technologies Group, Electronic Engineering Department, Universitat Politècnica de Catalunya—BarcelonaTech, 08034 Barcelona, Spain; vicente.jimenez@upc.edu (V.J.); sandra.bermejo@upc.edu (S.B.); joan.pons@upc.edu (J.P.-N.); 2 Centro de Astrobiologia (INTA-CSIC), 28850 Torrejón De Ardoz, Spain; manfredi@cab.inta-csic.es

**Keywords:** heat flux, regolith, thermal inertia, planetary exploration

## Abstract

The objective of this work is to present the first analytical and experimental results obtained with a 3D heat flux sensor for planetary regolith. The proposed structure, a sphere divided in four sectors, is sensible to heat flow magnitude and angle. Each sector includes a platinum resistor that is used both to sense its temperature and provide heating power. By operating the sectors at constant temperature, the sensor gives a response that is proportional to the heat flux vector in the regolith. The response of the sensor is therefore independent of the thermal conductivity of the regolith. A complete analytical solution of the response of the sensor is presented. The sensor may be used to provide information on the instantaneous local thermal environment surrounding a lander in planetary exploration or in small bodies like asteroids. To the best knowledge of the authors, this is the first sensor capable of measuring local 3D heat flux.

## 1. Introduction

Many physical and chemical processes are strongly dependent on thermal energy transfer manifested in temperature and heat flux changes. Temperature is a magnitude related with the system internal energy that can be easily measured using different devices: thermistors, thermocouples, semiconductor-based sensors or by monitoring IR radiation in contactless sensors, for example. Temperature changes are usually the result of changes in heat flux patterns. Heat flux is a measure of the thermal energy transfer associated with the conduction, convection and radiation mechanisms [1,2]. In some cases, other more complex phenomena such as phase changes [3,4], chemical reactions [5], or even magnetic effects [6] may also be involved. Measurement of heat flux is therefore one of the key physical parameters to monitor in many applications [7,8,9,10,11].

Additionally, heat thermal processes are governed by different equations depending on the specific mechanisms—Fourier law for heat conduction in solids, Stefan-Boltzmann law for radiation and other more exotic laws, such as for example the hyperbolic heat conduction (Cattaneo equation) for relativistic heat conduction. All these equations make use of isotropic thermophysical scalar constants (or their anisotropic versions [12]), which are material properties: thermal conductivity, heat capacity, emissitivity, and so forth. The characterization of the thermophysical properties of the materials themselves is therefore another area where heat flux is usually analyzed and/or measured [13,14,15]. The simultaneous measurement of temperature at localized points and heat flux offer a very good description of the local thermal processes taking place in a system.

Different sensing principles can be used to infer heat flux in surfaces, such as the detection of infrared radiation [16], contact thermopiles [17,18], indirect procedures such as calorimetry [19,20] or other approaches in which temperature gradients are measured and an estimation of the thermal conductivity is used [21].

One of the applications where the measurement of heat flux provides key information regarding geophysical processes, is in planetary exploration [22]. This measurement is related to the energy balance of all the processes occurring on the surface and subsurface of the planet. The annual, seasonal and diurnal variations of the surface temperature are tied to the properties of the upper few centimeters of the subsurface [23]. Heat flow, measured in the first centimeters of the regolith, therefore, can provide information on the heat transfer mechanisms involved in all these cycles, which in their turn depend on the thermophysical properties of the regolith, such as grain size [24], densification/compaction and possible atmospheric adsorption/desorption of gases [25], although this last factor may be small in some cases [26]. These phenomena may generate significant changes in the temperature-dependent thermal conductivity as a function of depth [27,28]. A complex history of depositional and excavational impact events may further induce additional inhomogeneities in the thermal properties of the regolith [29]. Inference of local 3D heat flux at different depths will help to understand the thermal context of the measurement and produce better estimations.

The corroboration of remote sensing predictions and models with in situ measurements is very important in order to ensure the quality of the predictions. This corroboration is common to many science objectives of many space missions. As an example, in situ thermal infrared observations on a C-type asteroid (Ryugu) have been recently made [30,31] in order to obtain, among other parameters, the thermal inertia of a boulder. It has been observed that a surface covered with such low-conductivity boulders would be compatible with remote measurements, whereas at the same time the measured thermal inertia was smaller than the values predicted from meteorites. In situ exploration is therefore needed to confirm and go beyond the limits of remote sensing, whenever possible.

The objective of this paper is to present the first 3D heat flux sensor for planetary regolith. Its structure is based on the work made by the authors on a miniaturized spherical 3D wind sensor for Mars atmosphere [32,33,34,35]. By operating the active parts of the sensor at constant temperature, it is possible to obtain sensor signals that depend on the vector heat flow at the sensor location, not on the temperature gradient. This opens the possibility of measuring heat fluxes without needing a prior inference of the thermal conductivity of the regolith. The fact that the sensor provides vector information opens also the possibility of ascertaining the local thermal context, analyzing possible horizontal geological inhomogeneities.

One of the parameters usually linked to surface heat flow is the thermal inertia [36], which is defined as $I=\sqrt{k\rho c}$, where *k* is the thermal conductivity, ρ is the density and *c* is the heat capacity. Unlike temperature, or other state variables, thermal inertia is an invariant surface property, which depends on the thermophysical properties of the subsurface materials [28].

The definition of thermal inertia comes from having a fractional thermal transfer function linking surface temperature and heat flux. In the case of an uni-dimensional, homogeneous and semi-infinite solid we have that [37]:

(1) $$J\left(t\right)=\sqrt{k\rho c}\frac{d^{\frac{1}{2}}}{dt^{\frac{1}{2}}}T\left(0,t\right),$$

where J(t) is the heat flux at the surface, $\frac{d^{0.5}}{dt^{0.5}}$ is a fractional derivative and T(z,t) is the temperature distribution (z=0 is the surface and increasing depth is increasing *z*). By assuming periodical sinusoidal variations, T(0,t)=Acos(ωt), we have [38]:

(2) $$J\left(t\right)=A\sqrt{k\rho c}\sqrt{\omega }cos\left(\omega t+\frac{\pi }{4}\right).$$

Inference of thermal inertia from remote sensing requires complex modeling of the layers in the soil, albedo, emissivity and other parameters. On the other hand, in situ measurement of heat flux, embedded in the first centimeters of the regolith and possibly at higher depths, provides better modeling of all these heat transfer processes [39].

If the heat flux measurement is made with enough accuracy and at a depth in which the diurnal, seasonal and annual cycles are not noticeable (at least at 50 cm from the surface in the lunar case for example, Reference [27,40]), then it is possible to infer the planetary heat flux. Typically this is done by measuring the temperature gradient as a function of depth, multiplying it by an estimation of the thermal conductivity of the regolith [27,41], although alternatively the use of thermopiles is being considered in some cases [42]. The estimation of planetary heat fluxes is a critical parameter to determine planetary thermal history models [43].

As it has been mentioned before, the objective of the paper is to present the structure of a 3D heat flow sensor for regolith. The sensor must be buried at the depth required by the specific target application, just as in the case of other heat flux sensors. Therefore, since the main heat transfer mechanism is conduction, it has been possible to obtain an analytical solution of a perfectly conducting sphere buried in the regolith. The paper is structured as follows. Section 2 describes the structure and basic operation of the sensor. Section 3 presents the analytical solution of the heat transfer problem. Section 4 presents and discusses the experimental results obtained. Finally Section 5 presents the conclusions.

## 2. Sensor Description and Operation

**Figure 1:**
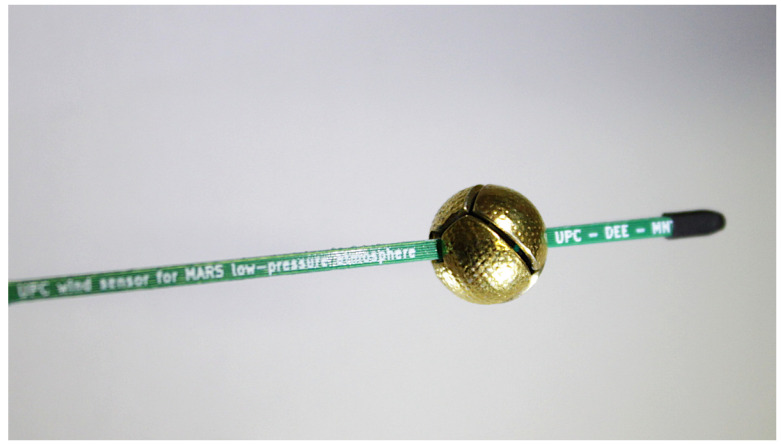
Photograph of the 4-sector spherical sensor (10 mm diameter), before being wrapped and inserted into the regolith simulant.

**Figure 2:**
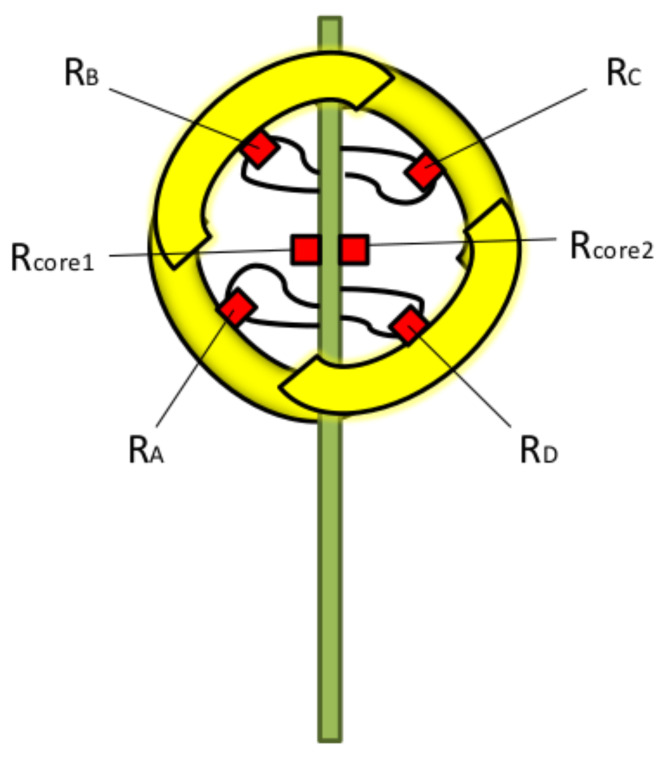
Schematic showing the placement of the Pt resistors associated with the four spherical sectors and cores.

As it has been mentioned in the Introduction, the sensor structure is the same proposed for 3D wind sensing on Mars [32,33,34,35]. The sensor is a 10 mm diameter sphere composed of four equally shaped sectors. The sectors are assembled to two superimposed printed circuit boards (PCBs). Each PCB provides mechanical support and signal routing for two sectors, as shown in Figure 1. A customized silicon die, manufactured in-house, which includes a Pt resistor, is attached to each sector in order to sense temperature and provide heating power. Finally, to control the temperature at the core of the sphere where the sectors are assembled, two additional dice are placed on the supporting PCBs, see Figure 2.

When working as a wind sensor, the structure is operated at constant temperature in all sectors and also in its core resistors. This temperature must be above that of the surrounding air. The heat transfer mechanisms in the sectors are: radiation, conduction and convection. The heaters associated to each sector must therefore provide the necessary power to compensate any variation in the thermal conductance of the sectors and possible external disturbances:

(3) $${\dot{Q}}_{\mathrm{surf}}={\dot{Q}}_{\mathrm{conv}}+{\dot{Q}}_{\mathrm{rad}}+{\dot{Q}}_{\mathrm{cond}},$$

where ${\dot{Q}}_{\mathrm{surf}}$ is the total power delivered to one sector heater, ${\dot{Q}}_{\mathrm{conv}}$ is the heat loss by convection, ${\dot{Q}}_{\mathrm{cond}}$ is the heat loss by conduction to the supporting structure and ${\dot{Q}}_{\mathrm{rad}}$ is the heat loss by radiation.

Radiation losses are minimized since a gold layer has been deposited on the surface of the sectors. Besides, conduction to the supporting structure is minimized by keeping the core at the same temperature of the sectors. Under these conditions, convection to the surrounding atmosphere is the main heat transfer mechanism in each sector, ${\dot{Q}}_{\mathrm{surf}}\approx {\dot{Q}}_{\mathrm{conv}}$. The heating power values delivered to each of the four heaters are the signals from which wind velocity can be inferred.

As commented above, this work investigates the possibility of using this same structure, buried now in the regolith, to measure the local heat flux. A protective thin plastic film has been applied to the surface of the sensor in order to prevent the regolith from entering the inner part of the structure. The sectors are made of silver, a metal with very high thermal conductivity, and kept at the same constant temperature. We will see in the next sections that any heat flux present in the regolith generates differences in the powers required in the sectors to keep their goal temperature constant. These differences will be proportional to the heat flux and independent of the thermal conductivity of the medium. In the next Section an analytical solution to this thermal problem is developed and discussed.

## 3. Analytical Solution of Heat Conduction in the Regolith

The objective of this section is to obtain the analytical solution of the Laplace equation of a metallic sphere embedded in a medium of constant thermal conductivity with an asymptotic vertical heat flux (in the z direction). This boundary condition is linked to having a uniform heat flux surrounding the sphere. Since the sphere is kept at constant temperature and no dissipation is made on the regolith, we have to solve the heat equation: $\nabla \cdot (k_{r}\nabla T)=0$, where $k_{r}$ is the thermal conductivity of the regolith, assumed to be constant.

The boundary conditions are:

(4a) $$\begin{array}{cc} T(r_{0},\theta )= & \delta T \end{array}$$

(4b) $$\begin{array}{cc} \lim_{r\to \infty }-k_{r}\nabla T & \to {\dot{Q}}_{0}\;\hat{z} \end{array}$$

(4c) $$\begin{array}{cc} \lim_{r\to \infty }T\left(r,\pi /2\right) & \to 0, \end{array}$$

where $r_{0}$ is the radius of the sphere and δT is the temperature on the surface of the sphere. We will consider, without loss of generality, that ${\dot{Q}}_{0}\hat{z}$, with ${\dot{Q}}_{0}\in R$, is the steady state heat flux in the surrounding regolith, far from the sphere. The third condition, (4c), ensures that the temperature at the surface of the sphere is δT above the temperature of the regolith at the height of the centre of the sphere, for r→∞.

The heat equation solution when imposing the above boundary conditions is:

(5) $$T\left(r,\theta \right)=\delta T\frac{r_{0}}{r}+\frac{{\dot{Q}}_{0}}{k_{r}}\left(\frac{r_{0}^{3}}{r^{2}}-r\right)cos\theta ,\;r\geq r_{0}$$

and the heat flow is then:

(6) $$\dot{Q}\left(r,\theta \right)=-k_{r}\nabla T=\left[k_{r}\delta T\frac{r_{0}}{r^{2}}+{\dot{Q}}_{0}\left(2\frac{r_{0}^{3}}{r^{3}}+1\right)cos\theta \right]\hat{r}+{\dot{Q}}_{0}\left(\frac{r_{0}^{3}}{r^{3}}-1\right)sin\theta \;\hat{\theta }.$$

This means that the heat flux on the sphere is normal ($\hat{n}=\hat{r}$) to its surface, as it would be expected from having a sphere of infinite thermal conductivity. Additionally, its magnitude is:

(7) $$\dot{Q}\cdot \hat{n}=k_{r}\delta T\frac{1}{r_{0}}+3{\dot{Q}}_{0}\;cos\theta .$$

Expression (7) indicates that the heat flux on the sphere presents two clearly distinct terms—one associated to the temperature set on the sphere, and basically due to the conduction through the regolith; and a second one, non-uniform, directly proportional to ${\dot{Q}}_{0}$. This second term, indicating a heat flux asymmetry on the sphere surface, is proportional to cosθ and is independent of the thermal conductivity of the regolith, $k_{r}$. Since the first term is an offset common to all sectors, if only the power differences between sectors are used, the sensor is indeed a heat flow sensor, and not a temperature gradient sensor. This is further emphasized by noting a very interesting property of the solution: the total power delivered to the surface of the sphere is constant for all values (and orientations) of the steady state heat flux. This value only depends on the first term of (7):

$${\dot{Q}}_{\mathrm{sph}}=4{\dot{Q}}_{\mathrm{avg}}=\;\int \int \;\dot{Q}\cdot \hat{n}\;dS=k_{r}\delta T4\pi r_{0},$$

where ${\dot{Q}}_{\mathrm{avg}}$ is the average power delivered to the sectors. This property will be used later in the derivation of a proper estimator of the heat flux in the regolith, independent of its thermal conductivity.

### 3.1. Properties of the Analytical Solution

**Figure 3:**
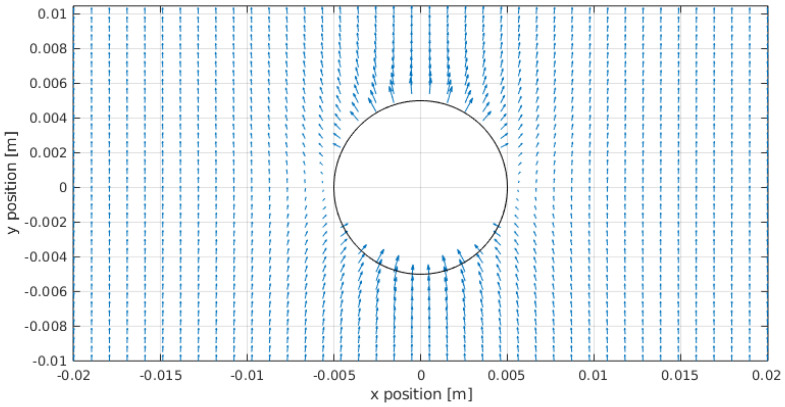
Heat flux on the complete domain for $k_{r}=0.1$ W/(mK), δT=0 K and ${\dot{Q}}_{0}=1$ W/m${}^{2}$. As it may be seen the $\dot{Q}\left(r\to \infty \right)\to {\dot{Q}}_{0}\hat{x}$. The bottom of the sphere is being heated whereas its upper part loses heat to the surrounding regolith. In this case, a heat flux in the $\hat{x}$ direction has been considered.

The first case we have analyzed is the distribution of the heat flux in the regolith and particularly on the surface of the sphere in the case δT=0. This would be the case of burying a uniform metallic sphere in the regolith, without applying any heating on it. Figure 3 shows the resulting heat flux. As it can be seen, the arrows depict an almost constant vertical heat flux at distances far from the sphere. This is consistent with the applied boundary condition of having $\dot{Q}\left(r,\theta \right)\to {\dot{Q}}_{0}\hat{z}$ for r→∞. On the other hand, at the north pole of the sphere, the heat flux is outgoing from the sphere (the sphere is providing heat to the regolith), whereas at the south pole the situation is reversed. This reflects the fact that, since there is a constant temperature condition on the surface of the sphere, the temperature gradient generates an asymmetry in the distribution of the heat fluxes, in the same way that non-constant charge distributions are obtained on the surface of a perfectly metallic sphere introduced in a constant electric field.

Since the sensor output signals are the values of the power delivered by the heaters, and therefore they cannot be negative, a δT>0 value is necessary to ensure that no heater is saturated to zero.

**Figure 4:**
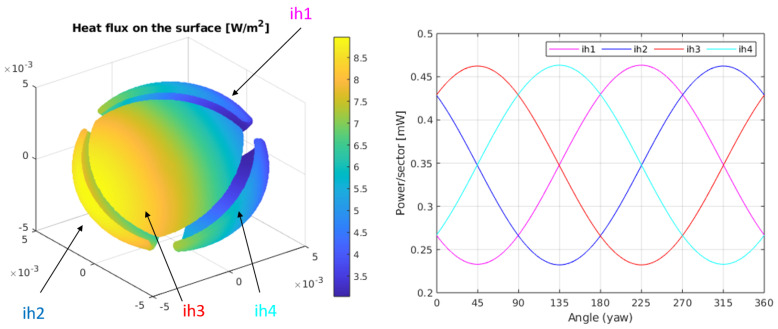
(**Left**) Heat flux on the surface of the sphere for $k_{r}=0.003$ W/(mK), δT=10 K, and ${\dot{Q}}_{0}$ = 1 W/m${}^{2}$. (**Right**) Integral of the heat fluxes on each of the sectors for the same conditions. In this case, a heat flux in the $\hat{x}$ direction has been considered.

Figure 4-Left shows the heat flux distribution on the surface of the sphere, for $k_{r}=0.003$ W/(mK), δT=10 K and ${\dot{Q}}_{0}=1$ W/m${}^{2}$. In this case, since δT>0 the heat flux is always positive (the sphere is at all points of the surface heating the regolith). Here, the z direction of the flux goes from left to right. Yaw rotations, therefore, generate a horizontal rotation of the heat flux on the sphere.

As we know from the analytical solution, the average value of the heat flux on the surface depends on δT and the thermal conductivity of the regolith, $k_{r}$. The information regarding the asymptotic heat flux on the regolith is included in the heat flux differences on the surface of the sphere. This is more clearly seen in Figure 4-Right where the integral of the heat flux on each sector is calculated when the sensor is rotated horizontally (yaw angle). The amplitude of the power differences between sectors will only depend on the value of the heat flux. The sectors have been numbered 1 to 4 and the corresponding surface integrals of the heat fluxes, over each sector, have been named as: ih1..ih4. The sectors have been associated in the left plot of the same figure with the corresponding integrated heat flux, using labels and arrows.

**Figure 5:**
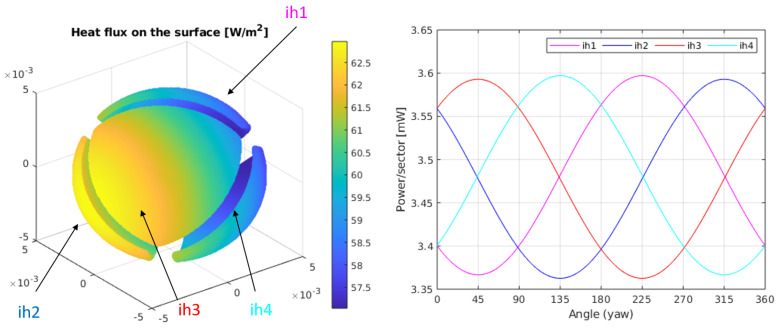
(**Left**) Heat flux on the surface of the sphere for $k_{r}=0.03$ W/(mK), δT=10 K, and ${\dot{Q}}_{0}$ = 1 W/m${}^{2}$. (**Right**) Integral of the heat fluxes on each of the sectors for the same conditions. In this case, a heat flux in the $\hat{x}$ direction has been considered.

Figure 5-Left shows the heat flux on the sphere for a case with identical conditions as those of Figure 4, but for a regolith with a thermal conductivity $k_{r}=0.03$ W/(mK). As it can be observed, the average heat flux on the sphere has increased substantially. This is something to be expected since the thermal conductivity has been increased by a factor 10. On the other hand, the difference between the maximum and minimum heat fluxes on the sphere are the same as before, since the constant multiplying the cosθ term in the solution does not depend on $k_{r}$. This phenomenon is reflected in Figure 5-Right, where the integral of the heat flux on each sector is calculated for a complete sweep of the yaw angle. The average power in each sector has been increased by a factor 10, in the same proportion as the conductivity of the regolith. On the other hand, the amplitudes of the power signals, as a function of the yaw angle, are the same for each sector. By subtracting their respective offsets, all curves in Figure 4 and Figure 5 perfectly overlap.

**Figure 6:**
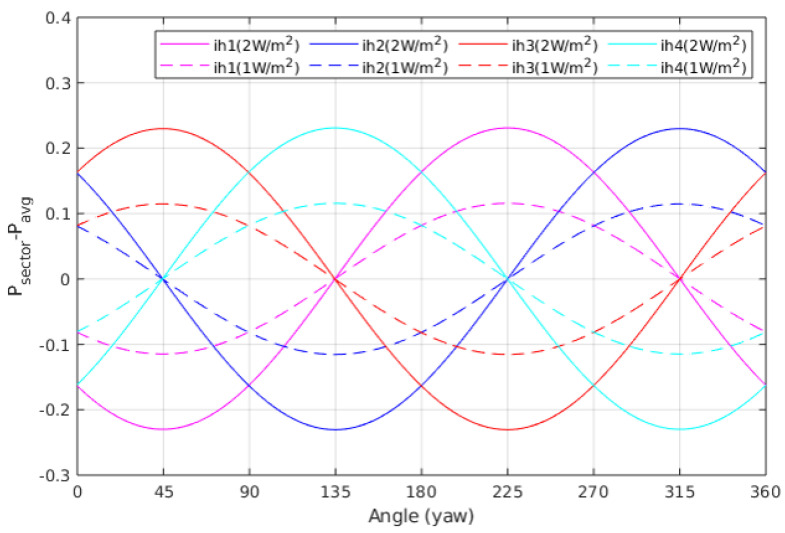
Power deviations from their average value of all sectors for the conditions in Figure 4 and Figure 5, and two heat flux values ${\dot{Q}}_{0}=1$ W/m${}^{2}$ (dotted lines) and ${\dot{Q}}_{0}=2$ W/m${}^{2}$ (continuous lines).

**Figure 7:**
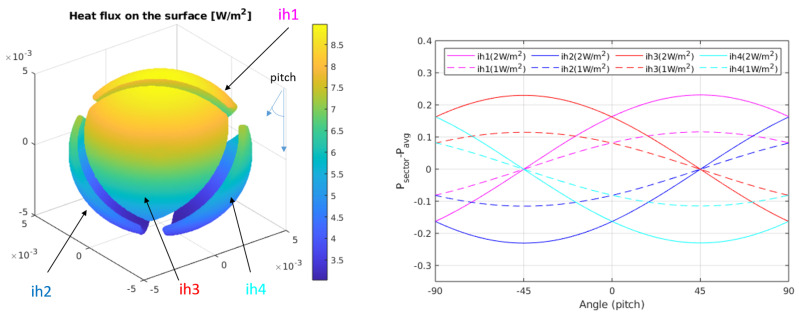
(**Left**) Heat flux on the surface of the sphere for $k_{r}=0.003$ W/(mK), δT=10 K and ${\dot{Q}}_{0}$ = 1 W/m${}^{2}$. (**Right**) Integral of the heat fluxes on each of the sectors for pitch rotations, for the same conditions. In this case, a heat flux in the $\hat{z}$ direction has been considered. This will be the configuration tested in the experimental results.

Since the main objective of this sensor is to provide a heat flux measurement, independent of the thermal conductivity of the regolith, it is necessary to work with the sector power differences, defined as $\Delta P_{i}=P_{i}-P_{\mathrm{avg}}$, where $P_{i}$ is the power delivered to sector *i* and $P_{\mathrm{avg}}=1/4\sum_{i}P_{i}$ is the average power in the sectors. Figure 6 and Figure 7 show the sector power differences, as a function of yaw and pitch rotations, respectively, for two different asymptotic heat flows (${\dot{Q}}_{0}=1$ W/m${}^{2}$ and ${\dot{Q}}_{0}=2$ W/m${}^{2}$). In both cases, from the amplitude of the signals it is possible to recover the magnitude of the heat flux, while from the relative values it is possible to obtain angle information.

### 3.2. Effect of Contact Resistance and Covering Film

As it has been previously mentioned, in the experimental setup the sensor has been protected by a plastic film before it has been buried in regolith. This could yield a thermal contact resistance between the surface of the sphere and the surrounding regolith. The effect generated in the sensor response can be taken into account in the previously obtained analytical solution, by considering two thermal conductivities in the medium surrounding the sphere:

- for $r_{0}<r<r_{1}$: the film (or thermal contact resistance) with a thermal conductivity, $k_{1}$, and
- for $r>r_{1}$: the thermal conductivity of the regolith, $k_{r}$.

The solution to this second geometry, considering the previous boundary conditions and continuity of temperature and heat flux at $r=r_{1}$ is:

(8a) $$\begin{array}{cc} T\left(r,\theta \right)=\frac{A_{0}}{r}+A_{1}+B_{1}\left(\frac{r_{0}^{3}}{r^{2}}-r\right)cos\theta , & \;r_{0}\leq r\leq r_{1} \end{array}$$

(8b) $$\begin{array}{cc} T\left(r,\theta \right)=\frac{A_{2}}{r}+\left(\frac{B_{2}}{r^{2}}-\frac{{\dot{Q}}_{0}}{k_{r}}r\right)cos\theta , & \;r_{1}\leq r, \end{array}$$

where:

(9a) $$\begin{array}{cc} A_{0} & =\frac{r_{1}}{\frac{k_{1}}{k_{r}}+\frac{r_{1}}{r_{0}}-1}\delta T \end{array}$$

(9b) $$\begin{array}{cc} A_{1} & =-\frac{A_{0}}{r_{0}}+\delta T \end{array}$$

(9c) $$\begin{array}{cc} A_{2} & =\frac{k_{1}}{k_{r}}, \end{array}$$

and

(10a) $$\begin{array}{cc} B_{2} & ={\left(\frac{1}{2r_{0}^{3}+r_{1}^{3}}\frac{2k_{r}}{k_{1}}+\frac{1}{r_{1}^{3}-r_{0}^{3}}\right)}^{-1}\cdot \left(\frac{r_{1}^{3}}{r_{1}^{3}-r_{0}^{3}}\frac{1}{k_{r}}-\frac{r_{1}^{3}}{k_{1}\left(2r_{0}^{3}+r_{1}^{3}\right)}\right)\cdot {\dot{Q}}_{0} \end{array}$$

(10b) $$\begin{array}{cc} B_{1} & =\frac{B_{2}}{r_{0}^{3}-r_{1}^{3}}-\frac{r_{1}^{3}}{r_{0}^{3}-r_{1}^{3}}\frac{1}{k_{r}}\cdot {\dot{Q}}_{0}. \end{array}$$

The heat flux in the isolating material ($r_{0}<r<r_{1}$) is then:

(11) $$\dot{Q}\left(r,\theta \right)=\left[k_{1}\delta T\frac{r_{0}}{r^{2}}+k_{1}B_{1}\left(\frac{2r_{0}^{3}}{r^{3}}+1\right)cos\theta \right]\;\hat{r}+B_{1}k_{1}\left(\frac{r_{0}^{3}}{r^{3}}-1\right)sin\theta \;\hat{\theta }$$

and in the regolith ($r>r_{1}$):

(12) $$\dot{Q}\left(r,\theta \right)=\left[k_{r}\frac{A_{2}}{r^{2}}+k_{r}\left(\frac{2B_{2}}{r^{3}}+\frac{{\dot{Q}}_{0}}{k_{2}}\right)cos\theta \right]\;\hat{r}+k_{r}\left(\frac{B_{2}}{r^{3}}-\frac{{\dot{Q}}_{0}}{k_{r}}\right)sin\theta \;\hat{\theta }.$$

Finally, on the surface of the sphere the heat flux is normal:

(13) $$\dot{Q}\left(r_{0},\theta \right)=\left[k_{1}\delta T\frac{1}{r_{0}}+3k_{1}B_{1}cos\theta \right]\;\hat{n}.$$

**Figure 8:**
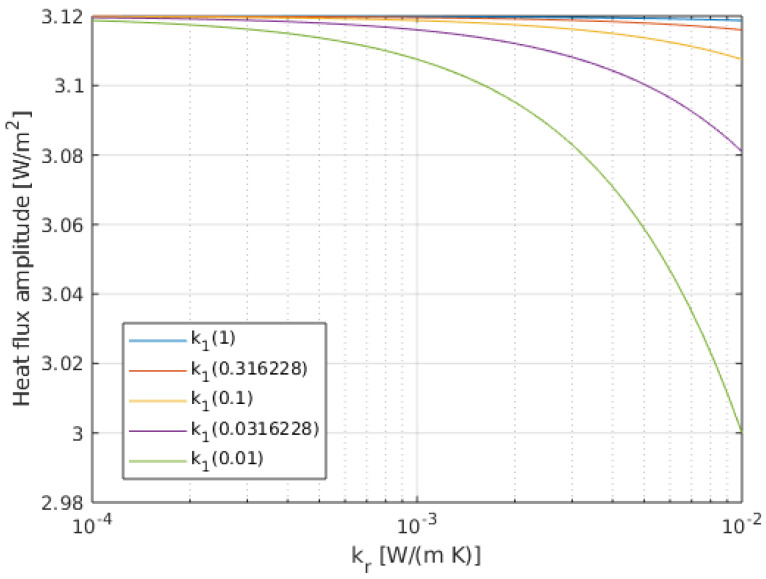
Amplitude of the heat flux variation on the surface, $3k_{1}B_{1}$, as a function of the conductivity of the regolith, for different values of the conductivity of the covering film, $k_{1}$. The film thickness is 100 μm and ${\dot{Q}}_{0}=1$ W/m${}^{2}$.

From Equation (13) it is clear now that, in this case, the amplitude of the cosθ term is no longer purely proportional to ${\dot{Q}}_{0}$. The influence of the thickness and thermal properties of the isolating layer must be analyzed by computing the associated expressions. Figure 8 presents the amplitude of the heat flux in-homogeneity (the $3k_{1}B_{1}$ term multiplying cosθ in the solution) as a function of the thermal conductivity of the regolith, $k_{r}$, for different values of the covering film thermal conductivity, $k_{1}$. The film thickness considered is 100 μm. We can conclude that the variation of the amplitude of the heat flux in-homogeneity for the swept conditions does not change beyond 4% from the ideal value.

## 4. Experimental Results

**Figure 9:**
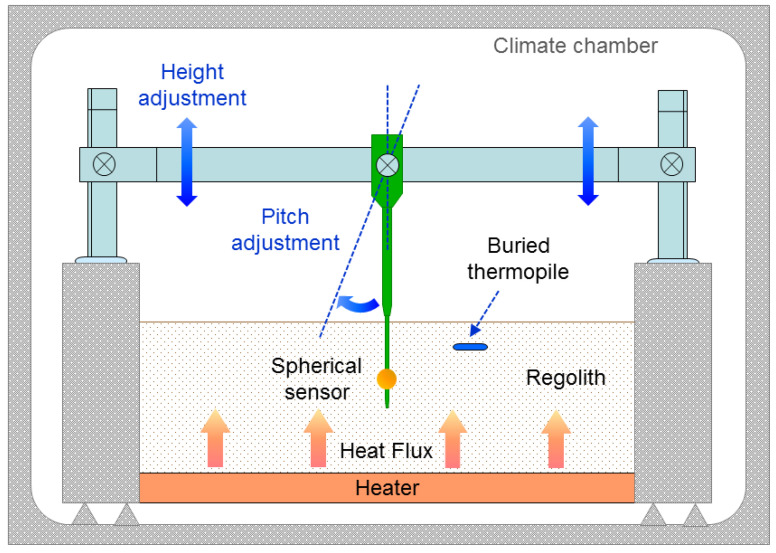
Schematic of the experimental setup. The complete structure has been placed in a climate chamber. The temperature of the bottom plate allows setting different heat fluxes in the regolith. The inner walls containing the regolith simulant are made of polystyrene.

A setup has been prepared in order to measure the response of the sensor to different heat flux conditions. Its scheme can be seen in Figure 9. A polystyrene structure has been built to house the regolith simulant, which is made of solid glass micro-beads with diameters in the range 40–70 μm and with a thermal conductivity of 0.1–0.2 W/(mK), depending on the porosity [44] (a more recent study of the thermal conductivity of powdered materials in vacuum can be found in Reference [45]). Thermal conductivity of granular media is clearly affected by pressure or lack of atmosphere.

The structure has a heater plate at the bottom that can be set at different target temperatures. The complete structure has been introduced inside a climate chamber, keeping the environment at constant temperature of 26 ${}^{\circ }$C. The heat flux near the surface of the regolith is measured by an auxiliary thermopile (GreenTeg gSKIN-XM-26-9C, with a 10 mm × 10 mm area), buried 1–2 mm below the surface. In this laboratory setup the sensors were not exposed to solar radiation and the climate chamber had controlled temperature at atmospheric pressure. Therefore, under steady state conditions, the heat flux measured by the thermopile near the surface of the glass micropowder and the heat flux at the sensor head should be very similar. The spherical sensor is suspended from a supporting structure that allows changing the height and angle inside the regolith.

**Figure 10:**
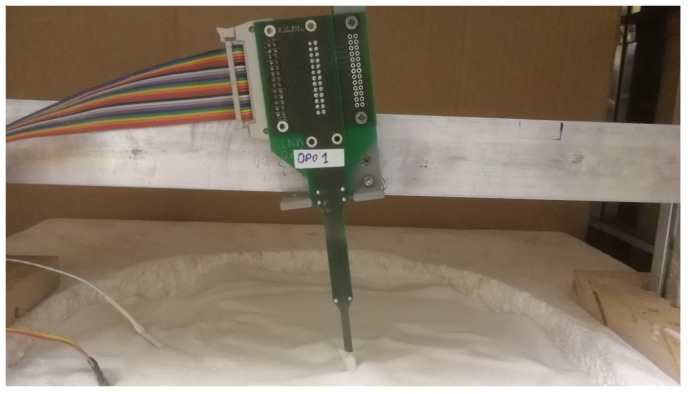
Sensor head buried in glass micro-beads.

As it has been mentioned before, the sensor has six custom silicon dice inside its spherical structure. Each die includes a platinum resistor. This way it is possible to monitor the temperature from its resistance value while applying any arbitrary power. An electronic setup implementing a thermal sigma-delta modulator allows setting the goal temperature using a feedback loop and monitoring the applied heating. The minimum power injected into each heater is $P_{\min}=2.8$ mW and the maximum $P_{\max}=31$ mW. The hot plate at the bottom of the setup is used to change the heat flux in the structure. A picture of the sensor head buried in the regolith simulant can be seen in Figure 10.

In order to observe the behaviour of the sensor, several experiments have been carried out. During the experiments, different parameters of the setup, such as the temperature of the bottom plate, or the angle of the sensor were changed at different points in time. Any adjustment triggers long transient responses, since the required temperature re-distributions are very slow. To observe the steady state response of the sensor, transients will be removed and only time intervals in which the sensor has achieved a constant response will be shown.

**Figure 11:**
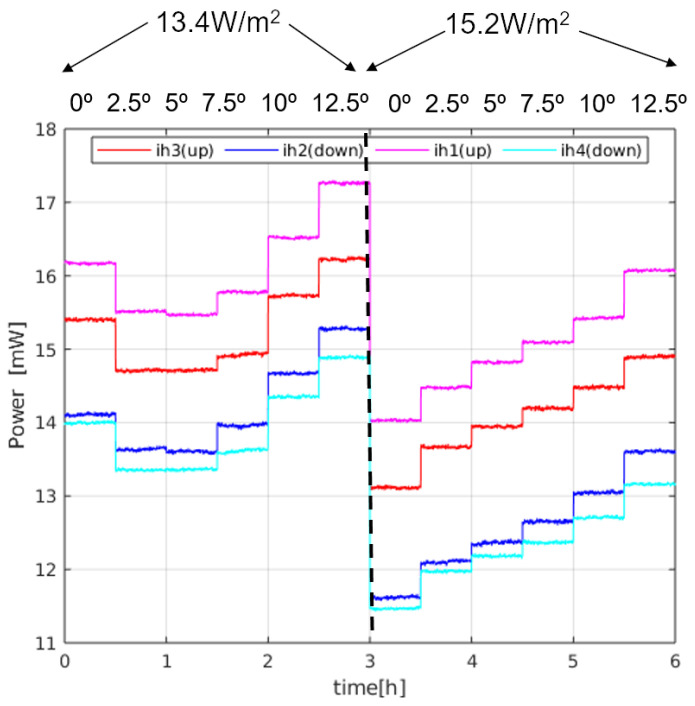
Sector powers in a 170 h experiment in which heat flux magnitude and angle are changed as a function of time. The plotted signals are the result of concatenating 30 min time intervals in which a steady state has been achieved. Note that height readjustment after angle change was made only after the first 3 angles during the first part of the experiment.

**Figure 12:**
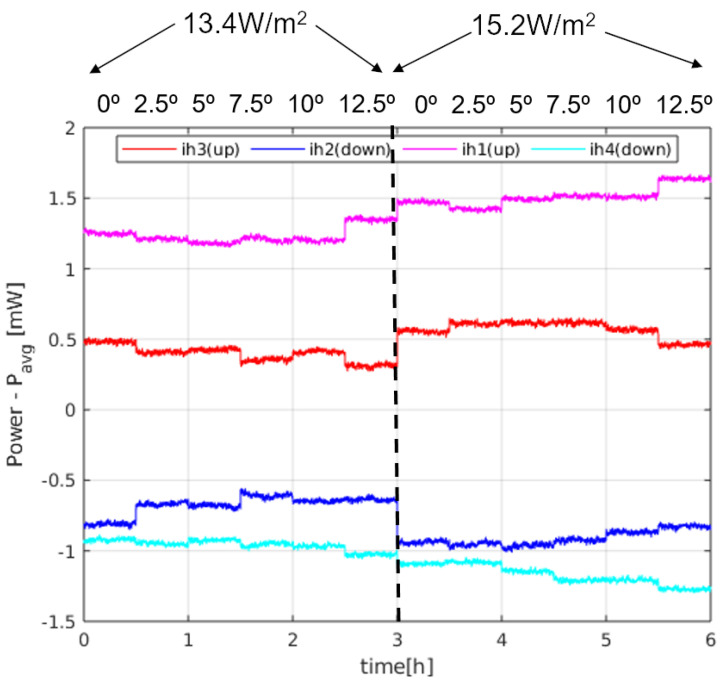
Sector power differences in the experiment of Figure 11.

**Figure 13:**
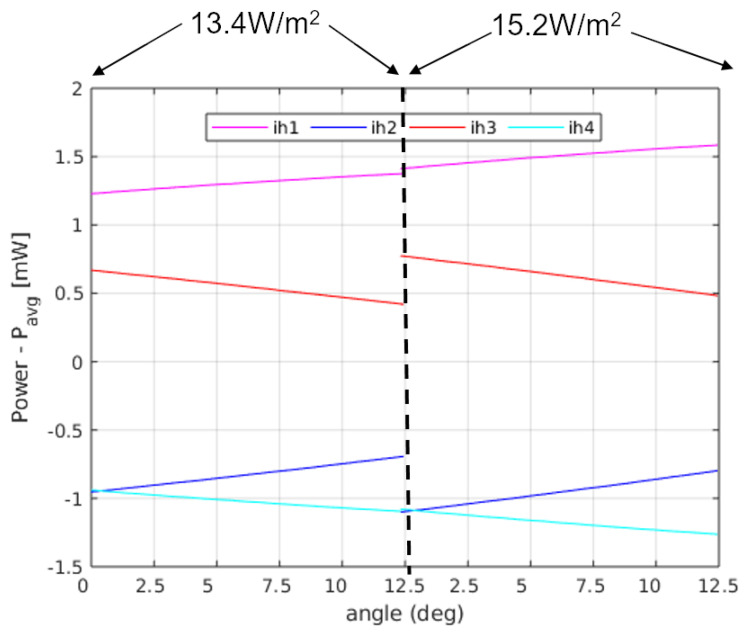
Theoretical prediction of the results in Figure 12, in case a misalignment of 11${}^{\circ }$ in the sector placement is allowed.

Figure 11 shows a sequence of steady state 30 min time intervals for an experiment in which the power of the sectors is monitored at different angles (0, 2.5${}^{\circ }$, 5${}^{\circ }$, 7.5${}^{\circ }$, 10${}^{\circ }$ and 12.5${}^{\circ }$) for two different temperatures of the bottom plate (28 ${}^{\circ }$C and 29 ${}^{\circ }$C), producing two different heat fluxes, as measured on the surface thermopile: 13.4 W/m${}^{2}$ and 15.2 W/m${}^{2}$. These heat fluxes have been measured at 0°. The heat flux introduced by the sensor itself is estimated to be 1.5 W/m${}^{2}$. Two main conclusions can be drawn from the measurements:

- First, the average power injected in the sphere as a function of pitch angle is 48–64 mW, which is within the range of the expected values predicted by the solution: $4\pi k_{r}\delta Tr_{0}$ = 37–88 mW for $k_{r}$ = 0.1–0.2 W/(mK) and δT = 6–6.5 ${}^{\circ }$C.
  It must be noted that the average power depends on the final height of the sensor with respect to the heater plate, within the regolith. Since the temperature of the sphere is kept constant at 35.5 ${}^{\circ }$C, depending on the resulting effective height after a pitch rotation, the temperature of the immediate regolith changes. We can observe this effect in Figure 11: in the first 3 intervals on the left, the effective height of the sphere was corrected after the corresponding changes in the angle, while that correction was dismissed in the rest of the intervals, to prevent slow temperature re-distributions in the regolith. As a consequence, in the second half of the experiment, for higher angles the effective height increases, which means that the temperature in the immediate regolith of the sensor decreases, and therefore more power is needed in the sectors.
- Second, the differences between the sectors grow as the heat flux increases. This effect is more clearly seen in Figure 12, where the difference between the power injected in the sectors and their average, $\Delta P_{i}$, is shown for all angles and both heat fluxes. The sector differences, $\Delta P_{i}$ increase for increasing flux.
  In order to see if Figure 12 matches the behaviour predicted by the theory, we may compare those with the results in Figure 7 for pitch angles between 0${}^{\circ }$ and 12.5${}^{\circ }$. The expected tendency is that the sectors in the upper position of the sphere (at 0${}^{\circ }$) have the same power (red and magenta lines), as well as the sectors at the lower position (blue and cyan lines) at 0${}^{\circ }$. For increasing angles, the sector powers separate (one increasing and the other decreasing). The main difference is that the magenta and red curves do not cross at 0${}^{\circ }$ pitch. However, if we apply the theoretical expressions for the experiment data, allowing an angle misalignment in the sectors of 11${}^{\circ }$, we obtain the theoretical solution seen in Figure 13. As it can be seen, the expected tendency, allowing for this adjustment, is very close to the experimental result. Other possible causes of this behaviour may be that the thin film wrapping the sphere is thicker near the north pole of the sphere. This may have generated a non-ideal behaviour for the upper sectors.

### Discussion

In this work we have used glass micro-beads as regolith simulant. It must be noted that there are many different types of regolith, and regolith simulants, and that further work is needed in order to optimize the procedure for placing the sensor inside the regolith. Additionally, we also think that further work is needed in order to characterize how the properties of the regolith may affect the measurement (for example cohesion, and possible anisotropies).

On the other hand, it has been possible to obtain an analytical solution of the idealized geometry of a perfectly conducting sphere embedded in a medium of constant thermal conductivity. The most relevant result of this analysis can be found in Equation (7), where the expression of the heat flux on the surface of the sphere is obtained. From the point of view of the analysis of the sensitivity of the sensor, a ’gain’ factor, *G*, can be defined as the factor between the external heat flux, ${\dot{Q}}_{0}$, and the amplitude of the asymmetry generated on the heat flux on the sphere, $3{\dot{Q}}_{0}\;cos\theta$. The value of this gain factor is therefore G=3. This parameter gives the idea of the heat flux imbalance generated on the sphere by any external heat flux.

Another sensitivity parameter can be found in Figure 7, for example, for the specific geometry of our sensor prototype. The power difference, with regard to the average generated in the sectors, generally depends on the angle but can be approximated by 0.1 mW/(W/m${}^{2}$) near a 0${}^{\circ }$ pitch. As an example, the heat fluxes measured by the Apollo 15 and 17 missions were 21 ± 3 mW/m${}^{2}$ and 15 ± 2 mW/m${}^{2}$, respectively [40]. These heat fluxes would produce power differences in the sectors of the order of 2.1 μW ± 0.3 μW and 1.5 μW ± 0.2 μW, respectively. We think that the measurement of these power magnitudes is difficult but achievable. Additionally, the inevitable tiny temperature differences between the sectors may generate power imbalances. In this regard, having a regolith with very low thermal conductivity, such as in the lunar case, will help to reduce the power differences generated in the sectors by errors in the sector temperatures. A more detailed error analysis is beyond the scope of the current paper, but certainly object of future work. More work is also needed in order to analyze the possibility of measuring the extremely small planetary heat fluxes and how the placement of the sensor, its supporting structure and the regolith properties will affect the quality of the measurement.

Finally, it must be pointed out that the sensor offers for the first time, to the best knowledge of the authors, the possibility of measuring local 3D heat flux using a miniature sensor that can be inserted in the regolith at an arbitrary angle (it does not require a specific orientation).

## 5. Conclusions

A spherical 3D heat flux sensor for planetary regolith has been presented. Since the sensor is buried in the regolith, the main heat transfer mechanism in the structure is conduction. We have obtained an analytical solution for the temperature and the heat flux vector field in the whole regolith domain. An asymptotic heat flux boundary condition is used to represent the heat flux in the area where the sensor has been buried. This condition forces an asymmetric distribution of the heat flux on the surface of the sphere. By operating all elements of the sphere at a constant temperature, differences in the heating signals, provided to keep all sectors at the same temperature, naturally arise.

The analytical solution found presents two distinct terms. The first one is a constant term added to all points of the surface. This term is proportional to the thermal conductivity of the regolith and to the temperature difference between the sphere and the average temperature of the immediate surrounding regolith. The second one, not dependent on the thermal conductivity of the regolith and proportional to the asymptotic heat flux in the regolith, generates a heat flux imbalance on the surface of the sphere with zero average. By using the power differences between different sectors of the sphere, or the differences with respect the average, an inference of the heat flux can be made, which is independent of the thermal conductivity of the regolith.

Experiments have been made using glass microspheres as regolith simulant. It has been shown that the power signals follow the behaviour predicted by the theory. In particular it has been observed that the average power delivered by the sectors depends, as expected, on the temperature of the surrounding regolith. Four signals have been used to infer the asymptotic heat flux—the differences between the power in each sector and their average instantaneous power. It has been shown that the signals change with angle and that the amplitude of these difference signals is proportional to the asymptotic heat flux.
